# Differences Between Lovastatin and Simvastatin Hydrolysis in Healthy Male and Female Volunteers: Gut Hydrolysis of Lovastatin is Twice that of Simvastatin

**DOI:** 10.1100/tsw.2003.121

**Published:** 2003-12-11

**Authors:** Tom B. Vree, Erik Dammers, Ivan Ulc, Stefan Horkovics-Kovats, Miroslav Ryska, IJsbrand Merkx

**Affiliations:** Institute for Anaesthesiology, University Medical Centre Nijmegen Sint Radboud, Nijmegen, The Netherlands

**Keywords:** lovastatin, simvastatin, statin-ß-hydroxy acid, pharmacokinetics, liver, gut, hydrolysis, plasma concentration, male-female differences

## Abstract

The aim of this pharmacokinetic evaluation was to show the effect of the extra methyl group in simvastatin on esterase hydrolysis between lovastatin and simvastatin in male and female volunteers. This study was based on the plasma concentration-time curves and the pharmacokinetics of lovastatin and simvastatin with its respective active metabolite statin-β-hydroxy acid obtained from two different bioequivalence studies, each with 18 females and 18 males. Results were: 1-The group of female volunteers showed a higher yield of the active metabolite β-hydroxy acid than the group of males (p < 0.002) for both lovastatin and simvastatin. This difference was not related to the body weight of both groups. 2-In the male/female groups, subject-dependent yield of active metabolite β-hydroxy acid was demonstrated, which was independent of the formulation. The variation in plasma/liver hydrolysis resulted in a fan-shaped distribution of data points when the AUC_t_ lovastatin was plotted vs. that of the β-hydroxy acid metabolite. In the fan of data points, subgroups could be distinguished, each showing a different regression line and with a different Y-intercept (AUC_tβ-hydroxy acid_). 3-Lovastatin hydrolysis was higher than simvastatin hydrolysis. 4-It was possible to discriminate between hydrolysis of both lovastatin and simvastatin by plasma/liver or tissue esterase activity.

The three subgroups of subjects (males/females) showing different but high yield of statin β-hydroxy acid can be explained by variable hydrolysis of plasma and hepatic microsomal and cytosolic carboxyesterase activity.

This study showed clearly that despite the subject-dependent hydrolysis of lovastatin/simvastatin to the active metabolite, males tend to hydrolyse less than females. The extra methyl group in simvastatin results in less hydrolysis due to steric hindrance.

## INTRODUCTION

The statins are reversible inhibitors of the microsomal enzyme HMG-CoA reductase, catalysing an early rate-limiting step in cholesterol biosynthesis, e.g., the conversion of HMG-CoA to mevalonate. Inhibition of HMG-CoA reductase by statins decreases intracellular cholesterol biosynthesis in liver and extrahepatic tissues[1,2,3,4,5,6,7,8].

Lovastatin (L) and simvastatin (S) in their lactone form are inactive; the active form is their metabolite statin-β-hydroxy acid (LA, SA) formed by hydrolysis by carboxyesterase activity in both plasma/liver and intestinal mucosa[9,10,11,12,13]. CYP 3A isoenzymes (CYP3A4) play an important role in the liver in the metabolism of lovastatin[11,14,15,16,17,18], as do CYP2D6 and CYP2C9[19]. In the intestines, CYP3A4 and CYP3A5 are the most abundant enzymes[10,20,21,22]. The major active metabolites found in human plasma are statin-β-hydroxy acid (LA, SA), 6'-hydroxy-, 6'-hydroxymethyl and 6'-exomethylene derivatives[14,23]. In humans, a linear increase in the inhibitory activity of statin (β-hydroxy acid) occurs in the dose range from 5–120 mg[12,24]. The reported elimination half-life of the active β-hydroxy acid is 1.9 h and the total body clearance is 31.8 L/h[25].

Lovastatin and simvastatin formulations are used in the treatment of elevated concentrations of total and LDL cholesterol, apolipoprotein B, and triglyceride in patients with primary hypercholesterolaemia, with combined hyperlipaemias (i.e., heterozygous familial hypercholesterolaemia, polygenic hypercholesterolaemia, familial dysbetalipoproteinaemia) and hypercholesterolaemias associated with other diseases like nephrotic syndrome, diabetes mellitus, and coronary artery disease[12,24].

Bioequivalence studies with 36 subjects (males/females) and a dose of 80 mg lovastatin or simvastatin revealed subject-related differences in the absorption, metabolism, and elimination of both lovastatin and simvastatin with its primary active metabolite statin-β-hydroxy acid[26,27]. The fixed dose for each subject results in different doses when expressed as dosage/body weight. Both males and females showed a variety in plasma/liver and tissue esterase activities as reported earlier for both compounds[26,27]. Simvastatin and lovastatin differ one methyl group in the molecular structure (Fig. 1).

The aim of this pharmacokinetic evaluation was to compare the effect of the extra methyl group in the structure of simvastatin on the hydrolysis by plasma/liver and tissue esterase activity, using the plasma concentration-time curves of lovastatin and simvastatin and its primary active metabolites.

## MATERIALS AND METHODS

The clinical trials were conducted by Cepha s.r.o., (CZ-323 13 Pilsen, Czech Republic) after granted approval by the Institutional Ethical Board.

### Subjects

Two different groups of 36 healthy Caucasians, 18 males and 18 females (nonpregnant, nonbreastfeeding), participated in two studies (simvastatin and lovastatin). Two male subjects withdrew from the simvastatin study (#9 due to health difficulties not related to simvastatin, and #25 due to business reasons). The demographic data are summarised in Table 1.

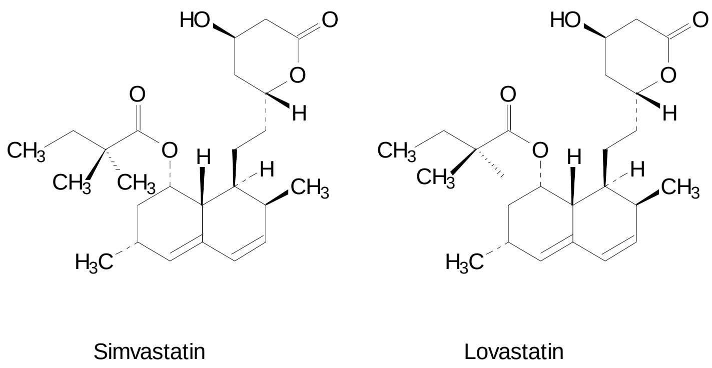

**FIGURE 1**. Lovastatin: [1S-[1α(R*),3α,7β,8β(2S*,4S*)8αβ]]-**2-methyl**butanoic acid, mp 174.5°C, $[\alpha]^{25}_D$ + 323°C, $C_{24}H_{36}O_5$, MW 404.55. Simvastatin: [1S-[1α,3α,7β,8β(2S*,4S*),8αβ]]-**2,2-dimethyl**butanoic acid, mp 135–138°C, $C_{25}H_{38}O_5$, MW 418.57.

**TABLE 1**
**Demographic Data of the Subjects**

| | n | Age (Years) | Body Weight (kg) | Height (cm) | Smoker (Yes/No) |
|---|---|---|---|---|---|
| **Lovastatin** | | | | | |
| All | 36 | 25.7 ± 5.5 | 70.5 ± 11.4 | 175 ± 10.7 | 5/36 |
| Males | 18 | 25.4 ± 5.2 | 77.7 ± 10.1 | 182 ± 8.7 | 5/18 |
| Females | 18 | 25.9 ± 5.9 | 63.2 ± 7.5 | 167 ± 5.9 | 0/18 |
| *p*, M/F | | 0.81 | *<0.001* | *<0.001* | |
| **Simvastatin** | | | | | |
| All | 36 | 23.6 ± 5.4 | 68.3 ± 11.5 | 175 ± 9.8 | 12/36 |
| Males | 16* | 23.0 ± 3.0 | 76.2 ± 8.1 | 183 ± 5.6 | 5/16 |
| Females | 18 | 24.2 ± 7.0 | 61.2 ± 9.4 | 168 ± 7.1 | 6/18 |
| *p*, M/F | | 0.54 | *<0.001* | *<0.001* | |
| ***p* Values lovastatin vs. simvastatin** | | | | | |
| All | 36 | 0.106 | 0.418 | 1.00 | |
| Males | 16* | 0.115 | 0.639 | 0.701 | |
| Females | 18 | 0.436 | 0.485 | 0.648 | |

* Two males discontinued the study.

## Experimental Design

Simvastatin — Participants were divided randomly into two groups with the aid of a computer-generated randomisation list. Group 1 was assigned to treatment sequence I-II (Formulation I-II). Group 2 was assigned to sequence II-I. During the two cross-over sessions, volunteers received each of the following treatments after an overnight fast, administered with 240 mL water:

- Formulation I = single oral dose of two simvastatin 40-mg film-coated tablets, batch number 65A (Simvastatin, Biochemie, A-6250 Kundl, Austria)
- Formulation II = single oral dose of two simvastatin 40-mg film-coated-tablets, batch number HJ28140 (Zocor®, Merck, Sharp & Dohme, Haar, Germany)

Lovastatin — Participants were divided randomly into two groups with the aid of a computer-generated randomisation list. Group 1 was assigned to treatment sequence I-II (Formulation I-II). Group 2 was assigned to sequence II-I. During the two cross-over sessions, volunteers received each of the following treatments after an overnight fast, administered with 240 mL water:

- Formulation III = single oral dose of two lovastatin 40-mg film-coated tablets, batch number 38 (Lovastatin, Biochemie, A-6250 Kundl, Austria)
- Formulation IV = single oral dose of two Lovastatin 40-mg film-coated tablets, batch number 0086100 (Mevinacor®, Merck, Sharp & Dohme, Germany)

Comparison of the studies was of parallel design.

## Drugs

- Lovastatin 40 mg, batch number 38 (Lovastatin, Biochemie, A-6250 Kundl, Austria), Formulation I
- Lovastatin 40 mg, batch number 0086100 (Mevinacor®, Merck, Sharp & Dohme, Germany), Formulation II
- Simvastatin 40-mg film-coated tablets, batch number 65A (Simvastatin, Biochemie, A-6250 Kundl, Austria)
- Simvastatin 40-mg film-coated tablets, batch number HJ28140 (Zocor®, Merck, Sharp & Dohme, Haar, Germany)

## Chemicals

Chemicals (pro analysis) were obtained from Baker, Merck, Fluka, and Sigma.

Simvastatin ([1*S*-[1α,3α,7β,8β(2*S**,4*S**),8αβ]]-2,2-**dimethyl**butanoic acid, mp 135–138°C, $C_{25}H_{38}O_5$, MW 418.57, CAS 79902-63-9, simvastatin-β-hydroxy acid ($C_{25}H_{40}O_6$, MW = 436.57); lovastatin (mevinolin, [1*S*-[1α(*R**),3α,7β,8β(2*S**,4*S**)8αβ]]-2-**methyl**butanoic acid, mp 174.5°C, $[\alpha]^{25}_{D}$ + 323°C, $C_{24}H_{36}O_5$, MW 404.55[1,6,28] and lovastatin β-hydroxy acid (mevinolinic acid) were obtained from Biochemie (Kundl, Austria).

## Trial Course

The treatment consisted of a single dose of two tablets each containing 40 mg statin administered orally with 240 mL of water in the morning of day 1 of each study period between 7:00 and 8:00, after an overnight fast. The subjects were not allowed to lie down or sleep for the first 3 h after dosing, to ensure normal absorption. If dizziness had occurred, the subjects would have permitted to lie down on their right side.

No alcohol-, caffeine-, xanthine-, or grapefruit-containing food or drink was allowed within 72 h before each dosing and during the confinement postdose periods.

From 48 h prior to each study period until 32 h after each study period, the intake of $CO_2$-containing beverages was prohibited, smoking was prohibited, and no strenuous activities were allowed 24 h before screening and follow-up examinations.

The subjects received 240 mL of water at dosing, and at 2 and 5 h after drug administration. Standardised meals with additional fluid (240 mL) were provided 10.5 h before dosing and at 4 h (+340 mL fluid), 6 h (+480 mL), 9 h (+240 mL), 12 h (+240 mL), 15 h (+240 mL), 25 h (+240 mL), and 29 h

(+480 mL) after dosing. The subjects were free to drink additional supplied beverages free of alcohol, $CO_2$, caffeine, and grapefruit from 6 h after drug administration.

## Blood Sampling

On the day of drug administration, between 6:00 and 6:50, an indwelling intravenous catheter (Vasocan Braunüle 20 G 11/4") was inserted into a forearm vein of each volunteer.

Blood samples (10 mL) were collected via the indwelling catheter in propylene tubes containing 0.20 mL of 5% $Na_2EDTA$ as anticoagulant. The indwelling catheter was flushed with 1–2 mL of a heparinised saline solution (250 I.U. of heparin in 100 mL of a 0.9% m/v NaCl solution) after each blood sample collection in order to maintain patency. 1–2 mL of blood was discarded before each blood sample drawn. After collection of the 16-h sample, the catheter was removed. The consecutive 2 samples were drawn by venepuncture.

The blood samples were collected at 0 h (predose) and at 0.33, 0.67, 1, 1,5, 2, 3, 4, 5, 6, 8, 10, 12, 14, 16, 24, and 32 h after dosing.

The blood samples were shaken gently, and centrifuged within 15 min after collection at 4000 rpm for 8 min at $4^oC$. The plasma samples were collected in three splits, capped, flash frozen and kept on dry ice until their storage in the freezer. The frozen plasma samples were stored in a freezer with $CO_2$ back-up at –75 ± $5^oC$ until their transportation on dry ice to the bioanalytical facility of Quinta-analytica and stored thereafter at –75 ± $5^oC$ until analysis.

## Bioanalysis

Lovastatin with its metabolite lovastatin-β-hydroxy acid and simvastatin with its metabolite simvastatin-β-hydroxy acid were analysed by means of a validated gas chromatography-mass spectrometry method (GC-MS, Quinta-analytica s.r.o., Hviezdoslavova 1600, CZ14900 Prague, Czech Republic) as described elsewhere[26].

## Pharmacokinetic Analysis

Pharmacokinetic parameters from the bioequivalence studies were calculated using the program Momanal 7.1 (Cepha).

The maximum plasma concentration ($C_{max}$, μg/L) and the time to reach peak concentration ($t_{max}$, h) were read directly from the plasma concentration-time curve.

The terminal half-life ($t_{1/2}$) was estimated from the slope (terminal rate constant $k_e$, $h^{-1}$) of linear regression of the semi-logarithmic plot of the terminal phase of the plasma concentration curve ($t_{1/2} = \ln2/k_e$), with the assumption is that the terminal phase was reached within the sampling period.

$AUC_t$ (μg.h/L) is the area under the plasma concentration-time curve and calculated by the linear trapezoidal rule from measured data points from time of administration until the time of the last measured concentration $C_t$.

$AUC_t$/kg is the $AUC_t$ corrected for body weight (μg.h/L/kg).

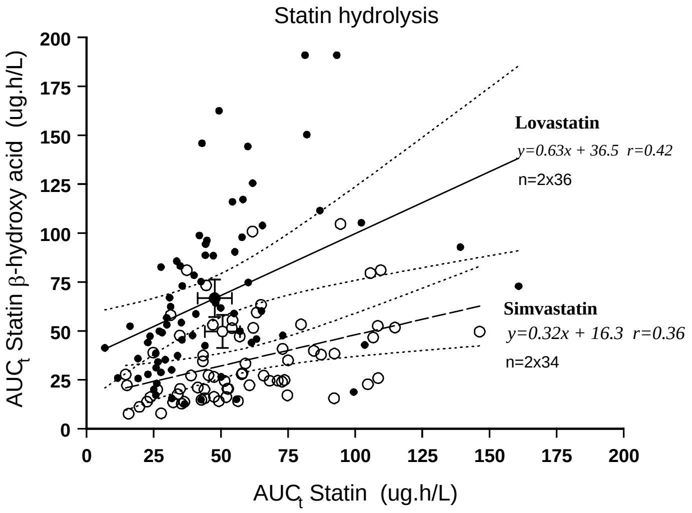

**FIGURE 2.** $AUC_t$ plots statin vs. statin β-hydroxy acid of lovastatin (solid dots, line) and simvastatin (open dots, interrupted line) with 95% CI intervals (dotted lines).

## Statistical Analysis

Analysis of variance (ANOVA, two-tailed, Gaussian distribution) was carried out according to standard procedures. Significance was defined at $p < 0.05$.

## RESULTS

The demographic data of the subjects (males and females) in both studies (simvastatin and lovastatin) were statistically identical, as shown in Table 1. The lovastatin and simvastatin dose in Formulations I and II were bioequivalent after single-dose administration under fasting conditions, based on the primary parameters $AUC_t$ and $C_{max}$ of the active moiety statin-β-hydroxy acid.

## Comparison Between Simvastatin and Lovastatin

The $AUC_t$ plots statin vs. statin-β-hydroxy acid of lovastatin and simvastatin look very similar as shown in Fig. 2, with the Y-intercept of the regression line of lovastatin being higher than that of simvastatin. The intercepts for all $AUC_t$ data of lovastatin and simvastatin are, respectively, 36.49 ± 1.051 and 16.26 ± 0.6909 (μg.h/L), $p < 0.0001$.

The slope of all lovastatin data, 0.6327 ± 0.1623, is higher than that of simvastatin, 0.3171 ± 0.0107 ($p < 0.0001$), which may indicate that the hydrolysis of lovastatin proceeds faster.

The mean $AUC_t$ values of the parent drug are similar: the $AUC_t$ lovastatin is 47.68 ± 3.249 (μg.h/L ±SEM, n = 72), the $AUC_t$ simvastatin is 50.56 ± 3.269 (μg.h/L, ±SEM, n = 68), $p = 0.5329$.

The mean $AUC_t$ values of the active metabolite statin-β-hydroxy acid differ significantly ($p = 0.0102$): the mean $AUC_t$ lovastatin-β-hydroxy acid is 66.65 ± 4.867 (μg.h/L, ±SEM, n = 72), the mean $AUC_t$ simvastatin-β-hydroxy acid is 49.72 ± 4.261 (μg.h/L, ±SEM, n = 68).

The conclusion must be that lovastatin hydrolyses 1.5 times faster than simvastatin.

## Male-Female Differences in Statin Hydrolysis

- Males — Fig. 3 shows the $AUC_t$/kg plots statin vs. statin-β-hydroxy acid of lovastatin and simvastatin in **males**. Three groups of subjects can be distinguished, who each have a particular slope and intercept of the regression line. In two groups, the Y-intercepts of lovastatin are higher than those of simvastatin, $p < 0.0001$; also the slopes of the regression lines in the same two groups males of lovastatin are higher than those of simvastatin, $p < 0.001$ (Table 2)**.** This may mean that the (gut) hydrolysis of lovastatin is twice as high as that of simvastatin.
- Females — Fig. 4 shows the $AUC_t$/kg plots statin vs. statin-β-hydroxy acid of lovastatin and simvastatin in **females**. Three groups of subjects can be distinguished, who each have a particular slope and intercept of the regression line. The Y-intercepts of lovastatin are higher than those of simvastatin, $p < 0.0064$ to $p < 0.0001$, while the slopes of the regression lines of the three groups are similar ($p$ = NS) (Table 3)**.** This may mean that the gut hydrolysis of lovastatin is twice as high as that of simvastatin.

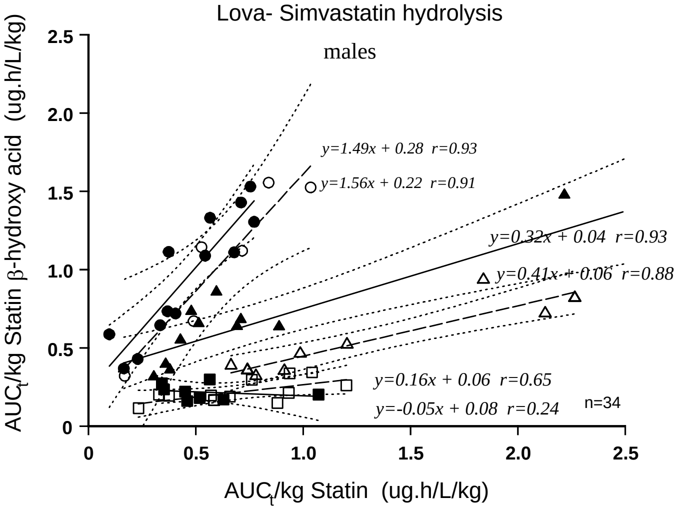

**FIGURE 3.** $AUC_t$/kg of lovastatin (solid markers, solid line) and simvastatin (open markers, interrupted line) plotted vs. the corresponding $AUC_t$/kg of the metabolite after an oral dose of 80 mg of the statin in **male** volunteers. Both statins show three groups of hydrolysis activities in both groups of male volunteers (parallel design). (Dotted lines are 95% CI).

**TABLE 2**
**Reduction of the Whole Data Fan (n = 2 × 34) in Males in Fig. 3 to Three Groups of Data, Simvastatin (S) and Lovastatin (L)**

| Regression | r | Y-intercept 95% CI | n |
|---|---|---|---|
| L, y = 1.56x + 0.22 | 0.91 | –0.015 to 0.48 | 14 |
| S, y = 1.49x + 0.28 | 0.93 | –0.43 to 0.66 | 6 |
| **Slope, *p* = 0.56** | | | |
| **Intercept, *p* = 0.13** | | | |
| L, y = 0.41x + 0.06 | 0.88 | 0.192 to 0.48 | 14 |
| S, y = 0.32x + 0.04 | 0.93 | –0.011 to 0.26 | 11 |
| **Slope, *p* = 0.0004** | | | |
| **Intercept, *p* < 0.0001** | | | |
| L, y = –0.05x + 0.08 | 0.24 | 0.12 to 0.37 | 8 |
| S, y = 0.16x + 0.06 | 0.65 | 0.013 to 0.20 | 13 |
| **Slope, *p* < 0.001** | | | |
| **Intercept, *p* < 0.001** | | | |

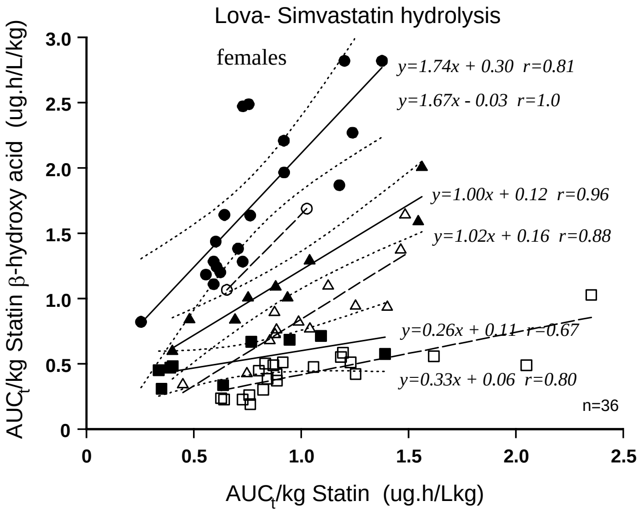

**FIGURE 4.** $AUC_t$/kg of lovastatin (solid markers, solid line) and simvastatin (open markers, interrupted line) plotted vs. the corresponding $AUC_t$/kg of the metabolite after an oral dose of 80 mg of the statin in **female** volunteers. Both statins show three groups of hydrolysis activities in both groups of female volunteers (parallel design). (Dotted lines are 95% CI).

## DISCUSSION

In this study we investigated the effect of the extra methyl group in simvastatin on the esterase activity of the statin to form the active statin-β-hydroxy acid. Lovastatin/Simvastatin must be regarded as pro-drugs, as -statin-β-hydroxy acid, and possibly more metabolites, are the active substances[7,9,12,16,17,23].

Subject-dependent yield of the primary active metabolite simvastatin- and lovastatin-β-hydroxy acid, in both females and males, independent of the formulation, was reported earlier[14,26,27].

**TABLE 3**
**Reduction of the Whole Data Fan (n = 2 × 36) in Females in Fig. 4 to Three Groups of Data, Simvastatin (S) and Lovastatin (L)**

| Regression | r | Y-intercept 95% CI | n |
|---|---|---|---|
| L, y = 1.74x + 0.30 | 0.81 | –0.17 to 0.91 | 19 |
| S, y = 1.67x – 0.03 | 1.0 | –0.026 to 0.003 | 2 |
| **Slope, *p* = 0.64** | | | |
| **Intercept, *p* = 0.0064** | | | |
| L, y = 1.00x + 0.12 | 0.96 | –0.0056 to 0.50 | 9 |
| S, y = 1.02x + 0.16 | 0.88 | –0.56 to 0.20 | 13 |
| **Slope, *p* = 0.73** | | | |
| **Intercept, *p* < 0.0001** | | | |
| L, y = 0.26x + 0.11 | 0.67 | 0.13 to 0.54 | 9 |
| S, y = 0.33x + 0.06 | 0.80 | –0.04 to 0.23 | 20 |
| **Slope, *p* = 0.055** | | | |
| **Intercept, *p* < 0.0001** | | | |

The higher AUC values in females of the active compound lovastatin-β-hydroxy acid can be the result of either a higher rate of hydrolysis or a lower rate of metabolism of lovastatin by cytochrome P450 (P3A4)[16,29,30]. Cheng et al. reported already higher mean steady-state plasma concentrations for simvastatin and lovastatin in female (20–50%) and elderly (40–60%) hypercholesterolemic patients (LDL >160 mg/dL; >4.14 mmol/L)[14]. The difference in $AUC_t$ values of active lovastatin between young females and males just reached significance ($p = 0.0451$). The present findings in healthy subjects and nondrug users indicate that the variation in AUC values and discrimination between the data of male and female subjects belong to the intrinsic pharmacokinetic behaviour of lovastatin and simvastatin. As the slopes of the regression lines of the groups of the highest $AUC_t$ lovastatin-β-hydroxy acid in males and females are similar (Figs. 3 and 4), the differences in the effect of sex hormones (and oral contraceptives) on the metabolism of statin and statin-β-hydroxy acid must be minimal[29,31,32,33]. With similar cytochrome P450 metabolism of lovastatin, the sex difference must affect the rate of hydrolysis caused by the differences in the esterase activities in plasma, liver microsomes, and cytosol[16,23]. These sex differences were not measurable for simvastatin after one dosage to healthy subjects[26,Vree personal observation] and after continuous administration in young and elderly patients[14]. Smith et al. reported the sex differences for fluvastatin, also females had higher AUC values than males[34]. This variation in plasma concentration and AUC values between males and females is much smaller than the increase in plasma concentration (+3×) by comedication with cytochrome P450 inhibitors such as gemfibrozil[35,36], erythromycin[37], verapamil[38], itraconazole[39], and the stimulator rifampicin (–10×)[40].

The variation in plasma/liver hydrolysis results in a fan-shaped distribution of data points when the $AUC_t$/kg statin is plotted vs. that of the metabolite, as shown in Figs. 2–4. In the fan of data points, subgroups could be distinguished, each showing a different regression line and with a different Y-intercept ($AUC_t/kg_{hydroxy\ acid}$). As shown earlier for the acetylation of mesalazine by the liver and by the gastrointestinal tract[41], it is possible to discriminate between hydrolysis of both lovastatin and simvastatin by plasma/hepatic or tissue esterase activity[26,27]. When there is only plasma/liver esterase activity responsible for the hydrolysis of -statin, then with $AUC_t/kg_{statin}$ approaching zero ($lim\ X \rightarrow 0$), the $AUC_t$/kg metabolite also must approach zero ($lim\ Y \rightarrow 0$), the 95% CI of the Y-intercept must contain the zero.

Both males and females show three subgroups of esterase activity to hydrolyse the statin to the -β-hydroxy acid. Lovastatin shows a higher rate of hydrolysis than simvastatin. This must mean that the extra methyl group at the C2 position in simvastatin must exert some sterical hindrance at the esterase. The difference in esterase is significant at the Y-intercepts in both males and females, suggesting that the gut hydrolyses lovastatin easier than simvastatin. **This makes the gut esterase susceptible for steric hindrance by the extra methyl group in simvastatin or the gut esterase differs significantly from the plasma/liver esterase.**

The effect of gut hydrolysis at the slope of the regression line disappears at the steeper slopes, indicating that liver hydrolysis overrules gut hydrolysis. Apparently gut and liver hydrolysis of these statins proceeds via different carboxyesterases. Simvastatin and lovastatin are also substrates for serum paraoxonase (PON1) isoenzymes and show stereospecificity[42].

Tang and Kalow investigated the nature of carboxyesterases and reported the existence of three esterases in man, one in plasma and two different in the liver[13]. The plasma esterase activity for the hydrolysis of lovastatin showed a 12-fold variation with three livers without any hydrolytic activity. The hepatic esterase activity was present in microsomes and cytosol. Thus, these *in vitro* results correspond with the *in vivo* results in the present study.

The Y-intercepts and regression lines in Figs. 3and 4 can be correlated by the three different esterase activities in plasma, liver microsomes, and cytosol. If there is no hepatic esterase capacity, then there is still plasma activity, resulting in a low yield of active metabolite lovastatin β-hydroxy acid. The ratio between hepatic activity in the microsomes and cytosol in the study of Tang and Kalow[13] result in similar figures in the present study, and gives a finer classification in metabolisers of lovastatin and simvastatin.

## Clinical Implication

This study showed clearly that females showed a higher yield of active metabolite than males, independent of the molecular difference in lovastatin/simvastatin. Both males and females showed three subgroups of subjects with high, medium, and low hydrolysis. The high metabolite yield can be attributed to plasma esterase plus hepatic microsomes and cytosol carboxyesterase activity. In contrast, more males than females showed extremely low yield of metabolite which may be attributed to sole plasma esterase activity or to one of the two other available mechanisms (liver/tissue). Males show a tendency to a lower rate of hydrolysis than females. Steric hindrance of the extra methyl group at C2 in simvastatin gives steric hindrance at the gut esterase enzymes. As a correlation between plasma concentration and enzyme activity of HMG-CoA inhibitory activity was demonstrated[16], the variation in plasma concentration is indicative for the variation in inhibitory activity and thus in effect. The dosage in males in general must be higher than the dosage in females, due to their lower rate of hydrolysis of lovastatin.

## BIOSKETCHES

**Tom B. Vree, Ph.D.,** Chemist and Pharmacologist at the University Medical Center Nijmegen, Nijmegen, Netherlands. His fields of interest include: drug analysis, drug metabolism, and pharmacokinetics. Dr. Vree is the author and/or coauthor of 600 scientific articles and 2 books.

**Erik Dammers**, Information Pharmacist, founded DADA Consultancy in 1984. DADA Consultancy has established a solid reputation for itself as a reliable and independent partner of international pharmaceutical companies. It offers a wide range of services for information scientists in the biomedical and regulatory disciplines. It has produced more than 1500 expert reports on a wide range of medical products and has an excellent track record of more than 90% successful submissions.

**Ivan Ulc, M.D., Ph.D.,** Director, Clinical Research, at CEPHA — Center for Pharmacology and Analysis, Pilsen, Czech Republic. Dr. Ulc has 84 publications, original scientific reports on clinical drug trials submitted to the Czech Regulatory Affairs Department, Ministry of Health, and other scientific contributions mostly on pharmacokinetic studies in man.
